# Neurobehavioral Function in School-Age Children Exposed to Manganese in Drinking Water

**DOI:** 10.1289/ehp.1307918

**Published:** 2014-09-26

**Authors:** Youssef Oulhote, Donna Mergler, Benoit Barbeau, David C. Bellinger, Thérèse Bouffard, Marie-Ève Brodeur, Dave Saint-Amour, Melissa Legrand, Sébastien Sauvé, Maryse F. Bouchard

**Affiliations:** 1 Department of Environmental and Occupational Health, Université de Montréal, Montréal, Québec, Canada; 2 CHU Sainte-Justine Mother and Child University Hospital Research Center, Montréal, Québec, Canada; 3 Centre for Interdisciplinary Studies in Biology, Health, Society and Environment (CINBIOSE), Université du Québec à Montréal, Montréal, Québec, Canada; 4 Department of Civil, Geological and Mining Engineering, École Polytechnique de Montréal, Montréal, Québec, Canada; 5 Department of Environmental Health, Harvard School of Public Health, Boston, Massachusetts, USA; 6 Department of Psychology, Université du Québec à Montréal, Montréal, Québec, Canada; 7 Department of Family Medicine, Ottawa Hospital, University of Ottawa, Ottawa, Ontario, Canada; 8 Department of Chemistry, Université de Montréal, Montréal, Québec, Canada

## Abstract

Background: Manganese neurotoxicity is well documented in individuals occupationally exposed to airborne particulates, but few data are available on risks from drinking-water exposure.

Objective: We examined associations of exposure from concentrations of manganese in water and hair with memory, attention, motor function, and parent- and teacher-reported hyperactive behaviors.

Methods: We recruited 375 children and measured manganese in home tap water (MnW) and hair (MnH). We estimated manganese intake from water ingestion. Using structural equation modeling, we estimated associations between neurobehavioral functions and MnH, MnW, and manganese intake from water. We evaluated exposure–response relationships using generalized additive models.

Results: After adjusting for potential confounders, a 1-SD increase in log_10_ MnH was associated with a significant difference of –24% (95% CI: –36, –12%) SD in memory and –25% (95% CI: –41, –9%) SD in attention. The relations between log_10_ MnH and poorer memory and attention were linear. A 1-SD increase in log_10_ MnW was associated with a significant difference of –14% (95% CI: –24, –4%) SD in memory, and this relation was nonlinear, with a steeper decline in performance at MnW > 100 μg/L. A 1-SD increase in log_10_ manganese intake from water was associated with a significant difference of –11% (95% CI: –21, –0.4%) SD in motor function. The relation between log_10_ manganese intake and poorer motor function was linear. There was no significant association between manganese exposure and hyperactivity.

Conclusion: Exposure to manganese in water was associated with poorer neurobehavioral performances in children, even at low levels commonly encountered in North America.

Citation: Oulhote Y, Mergler D, Barbeau B, Bellinger DC, Bouffard T, Brodeur ME, Saint-Amour D, Legrand M, Sauvé S, Bouchard MF. 2014. Neurobehavioral function in school-age children exposed to manganese in drinking water. Environ Health Perspect 122:1343–1350; http://dx.doi.org/10.1289/ehp.1307918

## Introduction

Manganese is widespread in the environment [Agency for Toxic Substances and Disease Registry (ATSDR) 2012]. It is an essential element, required for the function of many enzymes and involved in oxidative stress protection, as well as in the formation of connective tissue and bone. However, inhaled manganese is a potent neurotoxicant with well-documented effects in workplace settings (ATSDR 2012). Recent epidemiologic studies suggest that manganese exposure from drinking water is associated with poorer cognition and behavioral problems (Zoni and Lucchini 2013). High levels of manganese in water (MnW) are common in groundwater, because this element leaches from manganese-bearing minerals and rocks into the aquifers (Groschen et al. 2009).

Studies in Bangladesh among children exposed to high MnW reported associations with lower IQ scores (Wasserman et al. 2006), impaired perceptual reasoning and working memory (Wasserman et al. 2011), poorer academic achievement scores in mathematics (but not language) (Khan et al. 2012), as well as higher scores of internalizing and externalizing problems (Khan et al. 2011). In addition, we reported findings from two separate investigations conducted in Quebec (Canada) at MnW levels much lower than those in Bangladesh. In our pilot study, hair manganese levels (MnH) were associated with hyperactive and oppositional behaviors in children (Bouchard et al. 2007), and in the epidemiological study that followed, MnW was associated with lower IQ scores (Bouchard et al. 2011). On average, we estimated that there was a 6.2-point difference in IQ between children in the lowest and highest MnW quintiles.

A neurobehavioral test battery is often employed in studies aiming to identify neurotoxic effects of environmental exposures. This assessment typically results in a large number of scores used as outcomes, raising the problem of multiple testing. To avoid this issue, investigators often select a few test scores thought to be the most sensitive to detect neurotoxic effects associated with a given contaminant. This selection, however, is difficult because different studies often report slightly different findings. Although these apparent discrepancies could result from differences in the tests employed, they could also be attibutable partly to measurement errors in the neurobehavioral assessment. A better approach would be to consider test scores as measurements of an underlying neurobehavioral function, and use several scores as indicators for this function. Structural equation modeling (SEM) can be used to implement this approach, in which several different but related scores are used as indicators of a more global neurobehavioral domain, resulting in a more comprehensive assessment of the domain. In addition, this approach greatly reduces the number of outcomes (because several test scores are aggregated), therefore avoiding inference errors arising from multiple comparisons (Sanchez et al. 2005).

We conducted a cross-sectional study among 375 children exposed to a relatively wide range of MnW levels in Quebec (Canada), where the presence of manganese is naturally elevated. We previously reported lower IQ scores with higher MnW, MnH, and manganese intake from water ingestion (Bouchard et al. 2011). In the present study, we report the relation between these same manganese exposure indicators and neurobehavioral functions (memory, attention, motor function, and hyperactivity) in these children.

## Materials and Methods

*Study design and recruitment*. This cross-sectional study was conducted in eight municipalities located in southern Quebec (Canada) in 2007–2009. Municipalities were considered as potential study sites if their aqueduct was supplied by groundwater, and were selected to achieve a gradient of MnW. However, we did not limit our recruitment to children living in houses connected to the aqueduct, and many participating children lived in a house with a private well (we had no information on the MnW in private wells before recruitment). Children were recruited through elementary schools in the selected municipalities, and enrollment was restricted to children who had lived in the same house for > 3 months to ensure that the measured MnW was representative of the water children had been consuming for at least this duration. A total of 375 children 6–13 years of age participated in the study. The Human Research Ethics Board of the Université du Québec à Montréal approved the study protocol, and parents signed an informed consent.

*Manganese in water and hair*. We collected a water sample directly from the kitchen tap in each home. For homes that had a point-of-use filter attached to the tap, we collected one sample of filtered water, and a second sample with the filter removed. We used the following procedure to standardize tap water sampling (van den Hoven and Slaats 2006): *a*) open the tap for 5 min, *b*) close and leave untouched for 30 min, and *c*) collect first draw. We added 0.15 mL nitric acid (50%) to the 50-mL water sample and stored samples at 4°C. Manganese and other metals (arsenic, copper, lead, iron, and zinc) were measured by inductively coupled plasma mass spectrometry (ICP-MS) at the Environmental Chemistry Laboratory of McGill University (Montreal, Quebec, Canada). Further details about analytical techniques and quality assurance and control procedures can be found in the study by Barbeau et al. (2011).

For a subset of participants (*n* = 30 houses), we repeated tap water sampling four times (once per season) during 1 year to examine seasonal variability of MnW. Analysis of these data indicated an intraclass correlation coefficient of 0.91, indicating very little temporal variability in MnW concentrations (Bouchard et al. 2011).

Duplicate hair samples of approximately 20 mg were taken from the occiput of each child, cutting as close as possible to the root. We used the 2 cm closest to the scalp to measure manganese and other metals by ICP-MS in the laboratory of co-author S. Sauvé at Université de Montréal. Children were excluded from analysis when they reported using hair dye in the preceding 5 months because it could influence manganese hair content (Sky-Peck 1990). When manganese concentrations for certified hair material were outside of the designated concentrations, we excluded the measures from the analyses; we therefore had valid MnH measures for 313 children. When there was only one available measure (*n* = 45), due to contamination or insufficient hair for duplicates, the sole measure was retained for the statistical analyses; for the others, the mean of duplicates was used (*n* = 268). Further methodological details can be found in Supplemental Material, “Measurements of manganese hair concentration (Bouchard et al. 2011).”

*Manganese intake from water ingestion*. During the home visit, we orally administered a semiquantitative food frequency questionnaire to the parent and the child to assess manganese intake from water consumption, including direct water ingestion and water incorporated in food preparations (e.g., juices made from concentrate, soups) during the preceding month. We estimated water consumption from different sources—bottled, tap, tap filtered with a pitcher, and tap with an attached filter. For each water source, the amount consumed was multiplied by the measured or estimated concentration of manganese. We then divided the estimated intake by the weight of the child to derive an estimate of monthly intake in micrograms per kilogram. For untreated tap water and tap water treated with a carbon filter attached to the tap, the measured concentrations were used in the calculation. We conducted experiments to assess manganese removal efficacy for water filtered by a pitcher with activated carbon and an ion-exchange resin filter, showing an average manganese removal efficacy of 74% (Carrière et al. 2011), which we used in our calculations. For bottled water, we attributed a concentration of 0 μg/L based on a survey of bottled water in Canada that reported a median < 1 μg/L for manganese concentration (Dabeka et al. 2002).

*Assessment of neurobehavioral function*. Each child completed the following test battery: California Verbal Learning Test–Children’s Version (CVLT-C) (Delis et al. 1994), Conners’ Continuous Performance Test II Version 5 (CPT II) (Conners 2004), Digit Span, Santa Ana Test, and manual Fingertapping (Lafayette Instrument Company 2002). The Wechsler Abbreviated Scale of Intelligence (Wechsler 1999) was also administered, and the relation with manganese exposure was published elsewhere (Bouchard et al. 2011). The Conners’ Rating Scales were completed by a teacher (CRS-T) and a parent (CRS-P) (Conners 2000).

*Potential confounders*. We collected information from the mother on socioeconomic status indicators and other variables (e.g., alcohol and tobacco consumption during pregnancy), and we assessed maternal nonverbal intelligence with the Raven’s Progressive Matrices Test (Raven et al. 2003) and maternal symptoms of depression with the Beck Depression Inventory-II (BDI-II) (Beck et al. 1996). Potential confounders were chosen *a priori* based on established associations and/or plausible relations with the outcomes (Brooks-Gunn et al. 1996; Claus Henn et al. 2010; Neisser et al. 1996), and included child’s sex, age (years, continuous), maternal education (not completed high school/completed high school/some college/some university), nonverbal maternal intelligence (Raven score, continuous), family income (nine categories with Can$10,000 increment between two categories, entered as an ordinal variable), maternal depressive symptoms (Beck-II score, continuous), and lead concentration in tap water (log_10_ transformed). In addition, we used sensitivity analyses to assess the influence of additional adjustment for tap water arsenic concentration (log_10_ transformed), water source (aqueduct or private well), child’s test administrator, birth weight, and maternal smoking and alcohol consumption during pregnancy (entered one at the time in the models).

**Figure 1:**
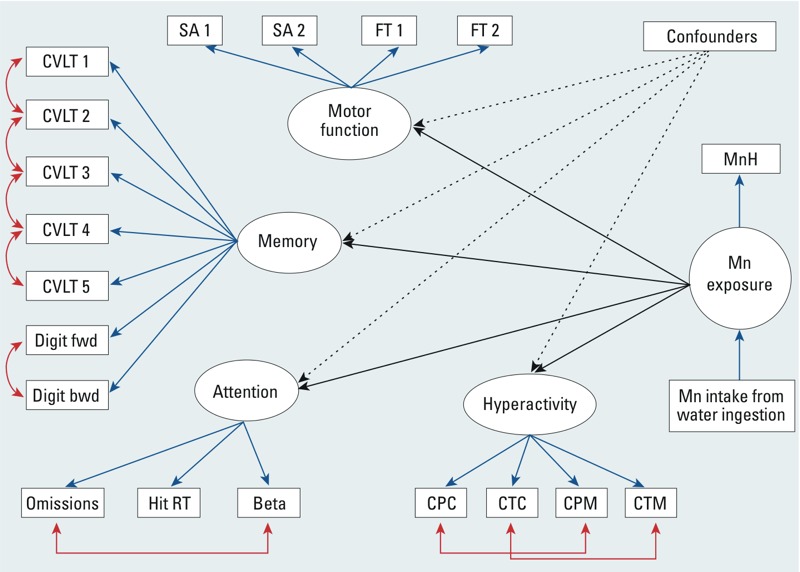
Conceptual path diagram for model 1 of the SEM for the associations between manganese exposure and neurobehavioral functions, with covariates adjustment. Manganese (Mn) exposure was modeled as a latent parameter indicated by hair manganese concentration (MnH), and influenced by manganese intake from water consumption. Four latent neurobehavioral functions were constructed: memory, attention, motor, and hyperactivity. The memory latent function was based on the number of correct responses on the learning trials of the CVLT–C for List A total trials 1–5 free recall (CVLT 1); List A, trial 1 free recall (CVLT 2); List A, trial 5 free recall (CVLT 3); Short delay free recall (CVLT 4); Long delay free recall (CVLT 5); Digit Span forward (Digit fwd) and backward (Digit bwd) scores. The attention latent variable was indicated by the scores of the CPT II test: number of failure to respond to target letters (omissions), overall hit reaction time (hit RT), and the response style indicator (beta). The attention test scores were reversed; therefore, higher attention scores indicate better performance. The motor function included scores on the Fingertapping [for dominant (FT 1) and nondominant (FT 2) hands] and the Santa Ana test [for dominant (SA 1) and nondominant (SA 2) hands]. The hyperactivity latent variable was indicated by parental (CPC) and teacher’s (CTC) hyperactivity and DSM-IV hyperactivity-impulsivity (CPM and CTM) scores from the Conners’ Rating Scales test. In models 2 and 3, the latent variable for manganese exposure was indicated solely by water manganese concentration, and manganese intake from water consumption, respectively.

*Statistical analysis*. We used SEM to examine relationships between exposure to manganese and children’s neurobehavioral functions (Sanchez et al. 2005). We built three models using different variables to quantify manganese exposure (Figure 1). First, we constructed a latent manganese exposure variable that is an underlying construct of measured MnH. In this model, we postulated that internal manganese loading is manifested by MnH and that this also depends on manganese intake from water ingestion. Second, given that there is no consensus that hair is a good biomarker of exposure to manganese (Eastman et al. 2013), we used MnW as the latent variable for manganese exposure. For the third model, we used the estimated intake of manganese from water ingestion as the indicator for the exposure latent variable to account for variation in the amount of water consumed by each child and the child’s weight.

Children’s scores on the neurobehavioral tests were used as indicators of four latent variables representative of underlying neurobehavioral domains: memory, attention, motor function, and hyperactivity (Figure 1). We constructed these latent variables using a confirmatory factor analysis allowing good discriminant validity between the factors. We also took into account the response modalities of outcome scores in accordance with SEM guidelines, especially for normality and the number of unique values (Kline 2011). To correct for local dependence when the correlation between the indicators could not be fully explained by the underlying latent variable, we allowed measurement errors of several outcomes indicating the same neurobehavioral function to correlate. We tested goodness of model fit using several indices (see Supplemental Material, Table S1).

The distributions of manganese exposure indicators (MnH, MnW, manganese intake from water consumption) and water lead concentrations were skewed, and were therefore log_10_ transformed. The results are presented as the adjusted change in the latent variables for neurobehavioral functions (β) associated with a 10-fold increase in manganese exposure indicators. Because β has no unit, we also present standardized association estimates, expressed as the change in percent of standard deviation (SD) of neurobehavioral function scores associated with a 1-SD increase in log_10_-transformed manganese exposure indicators. Some test scores were missing for some children: CPT II (*n* = 9), Digit Span (*n* = 14), Santa Ana (*n* = 2), CRS-P (*n* = 1), and CRS-T (*n* = 53). We imputed missing data using the full information maximum-likelihood method (Arbuckle 1996). In sensitivity analyses, we restricted our models to children with no missing data, and we included additional covariates in the models (listed in “Potential Confounders”). Finally, we explored effect modification by sex using multigroup SEM analyses.

Because SEM models data linearly, we also used generalized additive models (GAMs) with penalized smoothing regression splines to detect possible nonlinear relations. We extracted the scores from the factor-analytic component of the SEM and used them as outcome variables, adjusting for covariates. Manganese exposure indicators were introduced as spline functions. We examined departure from linearity using analysis of variance to assess the difference between the model with manganese levels introduced as a spline function and the model with manganese levels introduced as a linear term. To estimate thresholds for effect, we looked for the manganese level above which the estimated adjusted change in function was lower than the 95% confidence interval (CI) lower bound of the intercept (i.e., neurobehavioral score at the lowest manganese concentration).

All the outcomes variables were continuous; however, because some of them were not normally distributed, we used maximum-likelihood estimation with robust (Huber-White) standard errors (Huber 1967). The threshold for statistical significance was set to 0.05 (two-sided tests). We used the lavaan (Rosseel 2012) and mgcv (Wood 2011) packages in R (R Foundation for Statistical Computing, Vienna, Austria).

Finally, to assess the consistency of the SEM approach with a more traditional approach, we used general linear models to estimate the associations between manganese exposure indicators and individual test scores, adjusting for the same set of confounders.

## Results

*Descriptive statistics*. Table 1 shows the characteristics of children in the study, as well as levels of manganese exposure indicators. Almost all the children were white (99%) (data not shown), and 76% of mothers had at least some college education. Thirty-five percent of children resided in their current residence from birth, and most children (66%) had resided for > 5 years in their present home, based on residential history collected from parent. About half of participating children lived in a house connected to the aqueduct, and the other half in a house with a private well (51% and 49%, respectively). Tap water MnW ranged from 1 to 2,701 μg/L, with an arithmetic mean of 99 μg/L and a geometric mean (GM) of 20 μg/L, whereas estimated manganese intake from water ingestion ranged from 0 to 1,059 μg/kg/month with a GM of 5.5 μg/kg/month. There were 4.3% of homes with water levels of manganese above the U.S. health reference value of 300 μg/L [U.S. Environmental Protection Agency (EPA) 2003], and 43.7% exceeding the aesthetic level of 50 μg/L for manganese in drinking water (Health Canada 1979), above which the taste, smell, or color of water might be impaired. Water levels of arsenic and lead were elevated (≥ 10 μg/L) for 4% and 0.5% of children, respectively (data not shown). The Pearson correlation of MnW with other elements was 0.68 (iron), 0.26 (zinc), 0.11 (copper), 0.06 (arsenic), and –0.02 (lead).

**Table 1:** Manganese concentrations in hair and drinking water by characteristics of participants (Quebec, 2007–2009, children 6–13 years of age).

Characteristic	Water manganese			Hair manganese		
	*n* (%)	GM (μg/L)	*p*-Value^*a*^	*n* (%)	GM (μg/g)	*p*-Value^*a*^
Child
Sex			0.66			0.66
Male	175 (47)	19.1		149 (48)	0.75
Female	200 (53)	21.0		164 (52)	0.80
Age (years)			0.61			0.71
6–9	168 (45)	19.0		141 (45)	0.76
10–15	207 (55)	21.2		172 (55)	0.79
Total	375 (100)	20.1		313 (100)	0.78
Family^*b*^
Water source			< 0.001			0.02
Aqueduct	137 (53)	54.0		175 (56)	0.89
Private well	122 (47)	8.2		138 (44)	0.66
Family income			0.40			0.02
≤ Can$50,000	113 (44)	25.3		132 (42)	0.92
> Can$50,000	146 (56)	20.1		181 (58)	0.69
Maternal education^*c*^			0.61			0.99
Not completed high school	13 (5)	12.0		19 (6)	0.76
Completed high school	47 (18)	23.2		50 (16)	0.78
Some college	118 (46)	23.2		143 (46)	0.78
Some university	81 (31)	22.5		101 (32)	0.78
Nonverbal maternal intelligence (Raven)			0.84			0.04
< 23	98 (38)	24.6		111 (35)	0.75
23–25	97 (37)	21.2		124 (40)	0.68
> 25	64 (25)	20.5		78 (25)	1.01
Maternal depression (BDI-II)			0.29			0.31
Minimal (0–13)	233 (90)	21.3		290 (93)	0.77
Mild to severe (> 13)	26 (10)	33.1		23 (7)	0.97
Total	259 (100)	22.2		313 (100)	0.78
^***a***^Univariate analysis of variance and *t*-tests. ^***b***^One MnW measure per family (*n *= 259), one MnH measure per child (*n *= 313). ^***c***^In Quebec’s education system, students leave high school after grade 11, and enter postsecondary studies at the college level, as a prerequisite to university.						

In univariate analyses, there was no significant difference in MnW with respect to children’s sex and age, family income, maternal education, maternal depression, and maternal intelligence (Table 1). However, MnW was significantly higher in children living in homes connected to the aqueduct than in children living in homes with a private well (geometric mean of 54.0 vs. 8.2 μg/L, *p* < 0.001). Univariate associations with estimated manganese intake from water ingestion were consistent with those for MnW (data not shown).

Children’s MnH ranged from 0.1 to 20.7 μg/g with an arithmetic mean of 1.4 μg/g and a GM of 0.8 μg/g. In univariate analyses, MnH levels did not vary significantly with respect to children’s sex or age, or with maternal education or depressive symptoms (Table 1). However, MnH was higher in children living in homes connected to the aqueduct than in those living in homes with a private well, and MnH was also higher in children with family’s income ≤ Can$50,000 than in those with a higher income (both at *p* = 0.02). In addition, MnH differed significantly between maternal intelligence score categories (*p* = 0.04) (Table 1), but this association was no longer significant after additional adjustment for mother’s test administrator (*p* = 0.17) (data not shown).

*SEM*. Table 2 describes the factor loadings and estimated correlation of measured variables to each of the latent variables for neurobehavioral functions when MnH was the exposure (model 1). All test scores had good factor loadings and were adequate as indicators of latent variables, and this result was similar when exposure was based on MnW and estimated Mn intake from water ingestion (models 2 and 3, respectively; data not shown). Higher scores for memory, attention, and motor functions indicate better performance, but higher scores for hyperactivity suggest more problems related to hyperactivity.

**Table 2:** Factor loadings and estimated correlation of measured variables to the neurobehavioral function latent variables (Quebec, 2007–2009, children 6–13 years of age).

Latent variable, indicator	Factor loading	SE	*p*-Value	Percent of variance explained by latent construct
Memory
CVLT-C, List A total trials 1–5 free recall	1^*a*^	0	NA	0.75
CVLT-C, List A, trial 1 free recall	0.16	0.01	< 0.001	0.57
CVLT-C, List A, trial 5 free recall	0.22	0.01	< 0.001	0.70
CVLT-C, Short delay free recall	0.27	0.02	< 0.001	0.68
CVLT-C, Long delay free recall	0.26	0.02	< 0.001	0.70
Digit Span forward	0.12	0.02	< 0.001	0.50
Digit Span backward	0.10	0.01	< 0.001	0.54
Attention
CPT II, Omissions	1^*a*^	0	NA	0.47
CPT II, Hit RT	0.95	0.32	0.001	0.96
CPT II, Beta	0.34	0.12	0.005	0.29
Motor
Fingertapping, dominant hand	1^*a*^	0	NA	0.61
Fingertapping, nondominant hand	0.89	0.05	< 0.001	0.61
Santa Ana, dominant hand	0.33	0.04	< 0.001	0.72
Santa Ana, nondominant hand	0.28	0.04	< 0.001	0.71
Hyperactivity
CRS-P, Hyperactivity	1^*a*^	0	NA	0.86
CRS-P, DSM-IV: hyperactivity-impulsivity	0.91	0.05	< 0.001	0.82
CRS-T, Hyperactivity	0.49	0.19	0.01	0.48
CRS-T, DSM-IV: hyperactivity-impulsivity	0.57	0.22	0.008	0.53
Abbreviations: DSM-IV, *Diagnostic and Statistical Manual of Mental Disorders, Fourth Edition*;**NA, not applicable. ^***a***^For each neuro­behavioral function, the latent variable is constructed on the scale of the first component.				

In the first model, which was based on MnH, a 10-fold increase in MnH was significantly associated with differences of –3.6 points (95% CI: –5.2, –2.0) on the memory function and –4.2 points (95% CI: –6.7, –1.7) on the attention function (both at *p* < 0.01), after adjustment for confounders (Table 3). In standardized results, a 1-SD increase in log_10_-MnH was associated with lower memory (–24% SD; 95% CI: –36, –12%) and attention (–25% SD; 95% CI: –41, –9%) functions. No significant association was found between MnH and motor function (*p* = 0.57). The scores for hyperactivity tended to be lower (indicating fewer hyperactivity problems) for higher MnH with a difference of –2.0 points (95% CI: –4.6, 0.6; *p* = 0.13) for a 10-fold increase in MnH (Table 3).

**Table 3:** Differences in neurobehavioral function scores associated with manganese exposure indicators estimated by structural equation modeling (Quebec, 2007–2009, children 6–13 years of age).

Exposure indicator, neuro­behavioral function^*a*^	Difference in latent variable scores with 10-fold increase (95% CI)	Standardized difference in latent variable scores^*b*^ (95% CI)	*p*-Value
Model 1: MnH (*n* = 313)
Memory	–3.6 (–5.2, –2.0)	–24% (–36, –12%)	< 0.01
Attention	–4.2 (–6.7, –1.7)	–25% (–41, –9%)	< 0.01
Motor function	1.2 (–3.0, 5.4)	3% (–10, 16%)	0.57
Hyperactivity	–2.0 (–4.6, 0.6)	–10% (–24, 4%)	0.13
Model 2: MnW (*n* = 375)
Memory	–1.0 (–1.6, –0.4)	–14% (–24, –4%)	< 0.01
Attention	0.5 (–0.4, 1.3)	6% (–6, 18%)	0.31
Motor function	–1.2 (–2.7, 0.3)	–7% (–17, 3%)	0.11
Hyperactivity	–0.2 (–1.2, 0.8)	–2% (–11, 7%)	0.71
Model 3: manganese intake from water consumption (*n* = 375)
Memory	–0.4 (–0.9, 0.1)	–7% (–17, 3%)	0.13
Attention	0.1 (–0.6, 0.8)	2% (–11, 15%)	0.80
Motor function	–1.3 (–2.4, –0.2)	–11% (–21, –1%)	0.02
Hyperactivity	0.2 (–0.5, 0.9)	3% (–6, 9%)	0.51
All estimates were adjusted for child’s sex, age, maternal education, nonverbal maternal intelligence, family income, maternal depressive symptoms, and tap water lead concentrations. ^***a***^Higher scores for memory, attention, and motor functions indicate better performance, but higher scores for hyperactivity suggest more problems related to hyperactivity. ^***b***^Expressed as percent SD change in neuro­behavioral function for an increase of 1 SD in MnH, MnW, or manganese intake from water ingestion (the 3 manganese exposure indicators were log_10_-transformed).			

In the second model, where MnW was the exposure indicator, a 10-fold increase in MnW was associated with a significant difference of –1.0 point (95% CI: –1.6, –0.4; *p* < 0.01) of the memory function and a nonsignificant difference of –1.2 points (95% CI: –2.7, 0.3; *p* = 0.11) of the motor function. In standardized results, a 1-SD increase in log_10_-MnW was associated with memory function lower by –14% SD (95% CI: –24, –4%). No significant association was found between MnW and attention or hyperactivity functions.

The third model showed a significant association between estimated manganese intake from water consumption and motor function. A 10-fold increase in manganese intake was significantly associated with a difference of –1.3 points (95% CI: –2.4, –0.2; *p* = 0.02) on the motor function. In standardized results, a 1-SD increase in log_10_ manganese intake was associated with motor function lower by –11% SD (95% CI: –21, –0.4%). The scores for memory tended to be lower for higher manganese intake with a difference of –0.4 points (95% CI: –0.9, 0.1; *p* = 0.13) for a 10-fold increase in manganese intake from water ingestion (Table 3). We found no significant association between manganese intake from water consumption and and attention function or hyperactivity.

We assessed whether sex modifies the associations between manganese exposure indicators and neurobehavioral functions, and found that the association estimates for boys and girls were very similar (data not shown).

Separate models for each neurobehavioral function resulted in estimates similar to those of the model taking into account all four functions at the same time (data not shown). Likewise, the model including only children without missing data and the model with additional adjustment for water arsenic concentrations, birth weight, maternal smoking, and alcohol consumption during pregnancy resulted in very similar effect estimates (data not shown).

**Figure 2:**
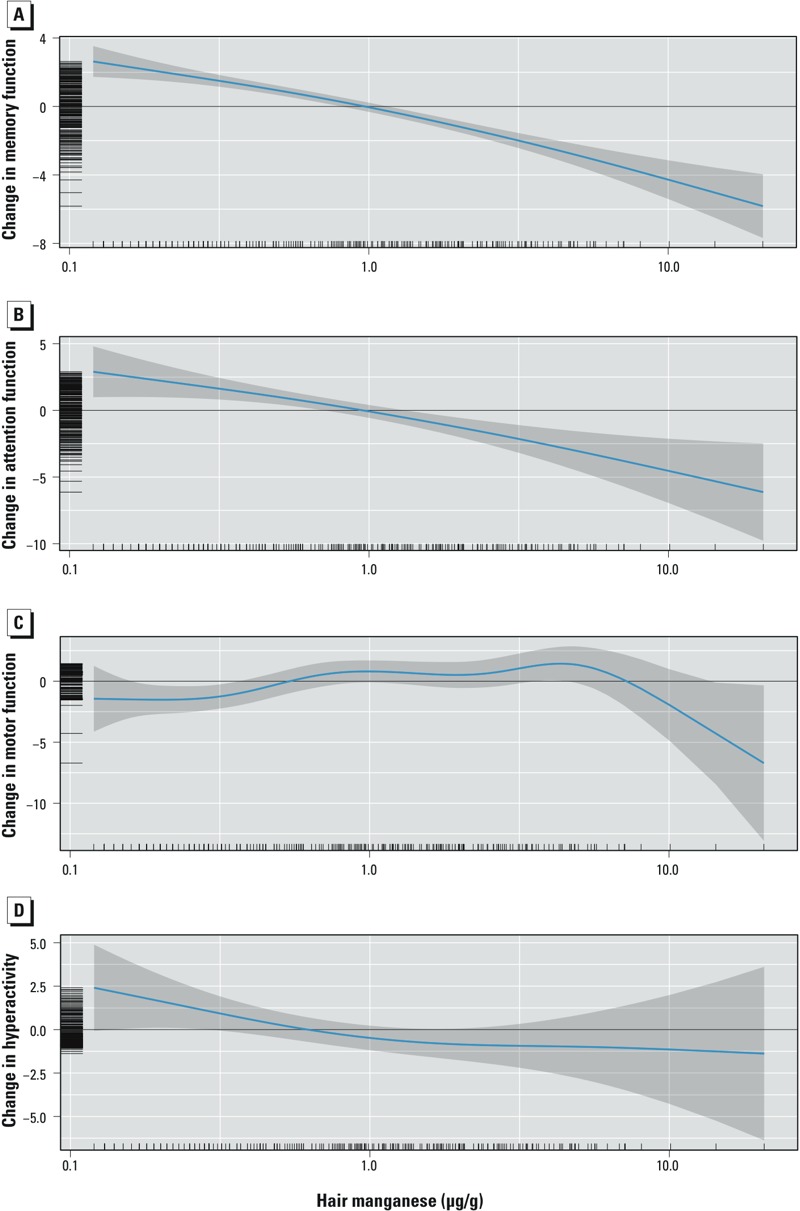
Adjusted associations between MnH concentration and memory (*A*), attention (*B*), motor functions (*C*), and hyperactivity (*D*). Covariates in models: child’s sex, age, maternal education, nonverbal maternal intelligence, family income, maternal depression, and water lead concentrations. Higher scores for memory, attention, and motor functions indicate better performance, but higher scores for hyperactivity suggest more problems related to hyperactivity.

**Figure 3:**
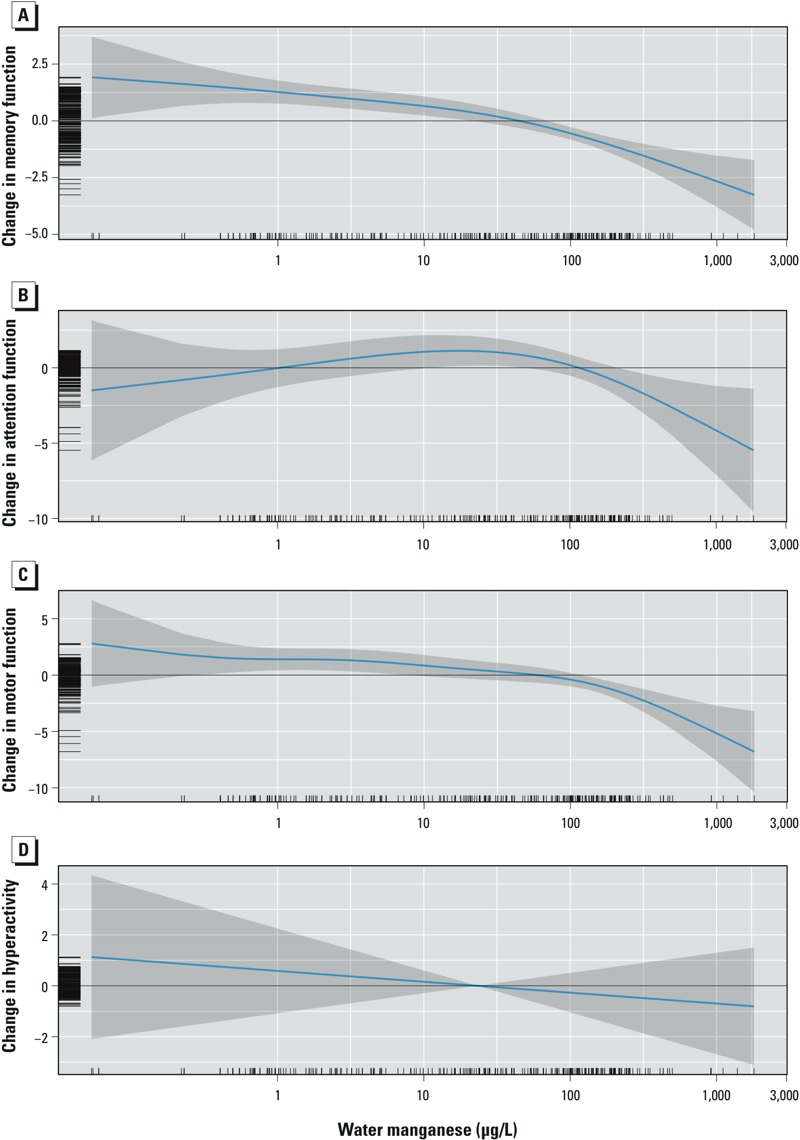
Adjusted association between MnW concentration and memory (*A*), attention (*B*), motor functions (*C*), and hyperactivity (*D*). Covariates in models: child’s sex, age, maternal education, nonverbal maternal intelligence, family income, maternal depression, and water lead concentrations. Higher scores for memory, attention, and motor functions indicate better performance, but higher scores for hyperactivity suggest more problems related to hyperactivity.

**Figure 4:**
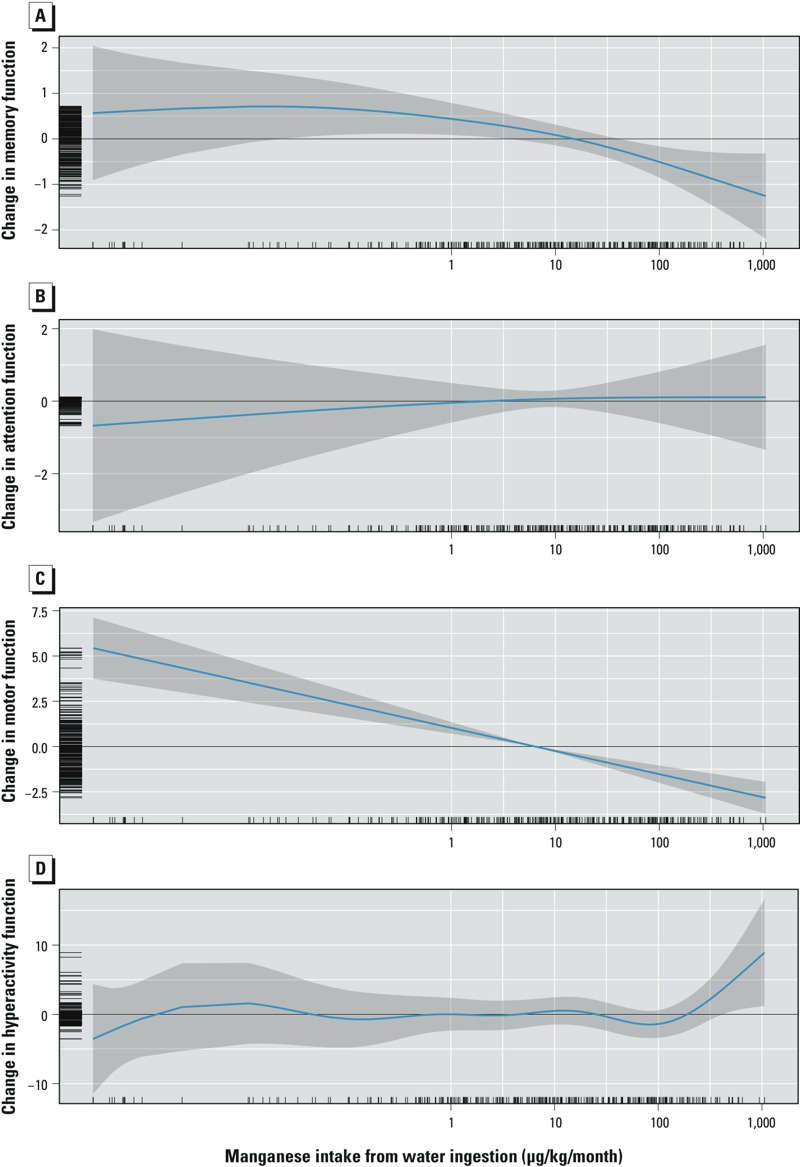
Adjusted association between manganese intake from water consumption and memory (*A*), attention (*B*), motor functions (*C*), and hyperactivity (*D*). Covariates in models: child’s sex, age, maternal education, nonverbal maternal intelligence, family income, maternal depression, and water lead concentrations. Higher scores for memory, attention, and motor functions indicate better performance, but higher scores for hyperactivity suggest more problems related to hyperactivity.

*GAMS to examine shape of the exposure–response relations*. We explored the shape of the exposure–response relation with GAMs for the constructed neurobehavioral functions, as shown in Figure 2 for MnH, Figure 3 for MnW, and Figure 4 for estimated manganese intake from water ingestion. MnH was significantly associated with poorer memory and attention (*p* < 0.001) with no significant departure from linearity (*p* = 0.1 for memory and attention; Figure 2A and 2B, respectively). For motor function, the association was significant (*p* = 0.02) and departed significantly from linearity (*p* < 0.01), with a slight increase at concentrations between 0.3 and 0.8 μg/g, and an apparent decrease in scores at MnH > 10 μg/g, but there were very few observations with such high levels (Figure 2C). The association for hyperactivity was not significant (*p* = 0.22) (Figure 2D).

In the GAM, children had significantly lower scores of memory with higher MnW (*p* < 0.001), and the relationship departed significantly from linearity (*p* = 0.045), with a steeper slope at levels > 100 μg/L than at lower concentrations (Figure 3A). No significant association was found between MnW and attention (*p* = 0.1) (Figure 3B). For motor function, the association with MnW was significant (*p* < 0.001), and departed significantly from linearity (*p* < 0.001), with a threshold indicating that scores decreased more steeply at concentrations above 180 μg/L (Figure 3C). No significant association was observed for hyperactivity (*p* = 0.47) (Figure 3D).

With GAM, estimated manganese intake from water ingestion was significantly associated with memory function (*p* = 0.038) in the GAM, and the function departed significantly from linearity (*p* = 0.04) (Figure 4A). No significant association was found for attention function (*p* = 0.88) (Figure 4B). For motor function, there was a significant association with manganese intake from water ingestion (*p* < 0.001) with no significant departure from linearity (*p* = 0.99) (Figure 4C). Finally, there was no significant association between manganese intake from water ingestion and hyperactivity (*p* = 0.44) (Figure 4D).

Supplemental Material, Table S2, shows estimated associations from general linear models of individual neurobehavioral test scores. The results were very consistent with the findings from SEM and GAM analyses.

## Discussion

We report that higher levels of exposure to manganese are associated with poorer performance of memory, attention, and motor functions, but not hyperactivity, in children. We estimated log-linear relationships between MnH and memory and attention functions and between manganese intake from water ingestion and motor function. The relationships for MnW exhibited a nonlinear shape, with steeper decreases in memory and motor functions at MnW levels > 100 and > 180 μg/L, respectively. These findings complement the previously reported association between the same three manganese exposure indicators (MnW, MnH, and manganese intake from water ingestion) with lower IQ scores in this same group of children (Bouchard et al. 2011). Furthermore, they are consistent with recent studies reporting adverse associations of manganese exposure with scores on tests of cognitive and motor functions (Zoni and Lucchini 2013). We did not observe a significant association with hyperactivity as previously reported (Bouchard et al. 2007; Khan et al. 2011), but MnW levels in the present study were considerably lower [GM for present study: 20 μg/L, compared with ≈ 300 μg/L (Bouchard et al. 2007), and ≈ 900 μg/L (Khan et al. 2011)].

Several mechanisms might underlie the association between manganese exposure and neurobehavioral outcomes. Studies have shown that manganese accumulates in the basal ganglia, white matter, and cortical structures (Guilarte et al. 2006). In animal models, manganese exposure disrupts the dopaminergic, glutamatergic, and serotonin systems (Moreno et al. 2009; Tran et al. 2002) that are essential to optimal cognitive functioning. Changes in gene expression (i.e., amyloid beta precursor-like protein) and markers of neurodegeneration in the frontal cortex (i.e., copper homeostasis dysregulation) induced by chronic manganese exposure may also underlie neurobehavioral changes (Guilarte et al. 2008; Schneider et al. 2009).

The present study has several strengths. We thoroughly assessed potential confounders, adjusting for several socioeconomic status indicators, as well as maternal intelligence and depression symptoms, and water lead concentrations. Furthermore, our results were robust to additional adjustment for water arsenic concentration, water source, child’s test administrator, birth weight, and maternal smoking and alcohol consumption during pregnancy. SEM allowed the simultaneous use of several indicators to better assess neurobehavioral functions, as well as integration of different exposure indicators (Budtz-Jørgensen et al. 2002). In addition, the SEM approach addresses issues arising from multiple testing and missing data that may not be adequately considered by standard regression analyses. The SEM findings were compared with the more traditional approach of analyzing each test score separately, and the findings were consistent.

There is no consensus on the best biomarker to assess exposure to manganese. Blood and urine manganese content have been shown to correlate poorly with manganese exposure (Smith et al. 2007). In population studies, MnH concentration has often been used as a biomarker of exposure. Significant correlations between exposure to manganese and MnH concentration have been reported in several studies (Agusa et al. 2006; Bader et al. 1999) including ours (Bouchard et al. 2007, 2011), supporting the contention that hair is a valid biomarker of manganese exposure. As the hair grows slowly, the composition reflects a time-weighted exposure over several months, which is appropriate since long-term exposure is postulated to cause neurotoxic effects. However, the use of hair as a biomarker has been criticized because of potential external contamination that is not removable by washing the samples before analysis (Eastman et al. 2013), which in the present case could result from bathing or showering in manganese-contaminated water.

There are several limitations to this study. The reported associations could be attributable to unmeasured confounders, but MnW did not vary with socioeconomic factors, thus reducing the potential for confounding. Nonetheless, possible residual or additional unmeasured confounding by other factors cannot be ruled out. However, our findings are not likely to be explained by anthropogenic contaminants, because in our study area, the contamination of water by manganese results from natural processes associated with the bedrock geology, not human activities. There are no industrial sources of manganese emission in the study area, and the gasoline additive methylcyclopentadienyl manganese tricarbonyl (MMT) has not been used in Canada since 2004 (Finkelstein and Jerrett 2007). Bolté et al. (2004) showed very low atmospheric manganese concentrations in rural areas of Quebec, with a mean of 0.005 μg/m^3;^ this is 10 times lower than the U.S. EPA inhalation reference concentration of 0.05 μg/m^3^ (U.S. EPA 1993).

The cross-sectional design of the study limits the ability to draw strong causal inferences, although reverse causality is improbable. It is not known whether exposure during a critical developmental period is responsible for our observations. The majority (66%) of participating children had been living in the same home for at least 5 years; therefore, exposure levels are representative of several years. Finally, our assessment of children’s exposure to manganese from drinking water was based on a single measurement; however, repeated measurements in the same houses showed very little time variability in MnW levels, suggesting that the value measured on one occasion is representative of long-term exposure (Barbeau et al. 2011).

## Conclusion

The World Health Organization (WHO) has recently discontinued its 400-μg/L guideline for manganese in drinking water, arguing that “this health based value is well above concentrations of manganese normally found in drinking water” (WHO 2011). In fact, drinking-water supplies with manganese concentrations exceeding this level are found in many countries, exposing tens of millions of people worldwide (Frisbie et al. 2012). Coupled with our previous report of MnW-associated IQ deficits (Bouchard et al. 2011), the present findings suggest the potential for harmful effects at levels commonly encountered in groundwater.

## Supplemental Material

(331 KB) PDF
