# Supplementary material for: Neurobehavioral Function in School-Age Children Exposed to Manganese in Drinking Water
Source: Environ Health Perspect. 2014 Sep 26;122(12):1343–50. doi: 10.1289/ehp.1307918 (PMC4256698; doi:10.1289/ehp.1307918)
Supplement: (331 KB) PDF [file ehp.1307918.s001.508.pdf]

Environmental Health Perspectives doi:10.1289/ehp.1307918

Supplemental Material

Neurobehavioral Function in School-Age Children Exposed to Manganese in Drinking Water

Youssef Oulhote, Donna Mergler, Benoit Barbeau, David C. Bellinger, Thérèse Bouffard, Marie-Ève Brodeur, Dave Saint-Amour, Melissa Legrand, Sébastien Sauvé, and Maryse F. Bouchard

Measurements of manganese hair concentration (Bouchard et al. 2011)

Hair samples were cleaned by sonication for 15 min in 20 mL of 1% Triton X-100 solution in a 50-mL beaker, rinsed 3 times with distilled milliQ water, and dried in a convection oven at 70 °C for 24 h. Duplicate samples of approximately 20 mg were weighed and digested with 1 mL of concentrated nitric acid and 1 mL of hydrogen peroxide (30% Suprapur®), in 7 mL teflon vials for 24 h. Samples were filtered with filter paper (Fisher Scientific, Q5), and the volume was completed with distilled-deionised water (Millipore Ultra Pure Water System, 18 mΩ•cm) to 10 mL. Reagent blanks and certified hair material (GBW 09101, Shanghai Institute of Nuclear Research) were incorporated into the preparation of each set of hair samples. Trace metal analysis (Mn, Pb, Fe, As, Zn, and Cu) was performed by inductively coupled plasma mass spectrometry (Varian ICP-820 MS). Calibration curves were run every 30 samples, together with laboratory blanks. When manganese concentrations for certified hair material were outside of the designated concentrations, the hair samples of the corresponding set were excluded from the present statistical analyses. The duplicates were highly correlated ($n = 268$ pairs; Pearson $R = 0.97$).

Table S1. Model fit indices[a] for SEM analyses.

Exposure indicator, neurobehavioral function	p-value (chi-square)	CFI	RMSEA (90% CI)	SRMR
Model 1: Hair manganese (n=313)				
Memory	0.29	0.997	0.017 (0.000-0.039)	0.026
Attention	0.10	0.953	0.036 (0.000-0.063)	0.029
Motor	0.003	0.969	0.056 (0.036-0.076)	0.039
Hyperactivity	0.28	0.995	0.027 (0.000-0.052)	0.031
Model 2: Water manganese (n=375)				
Memory	0.55	1.000	0.000 (0.000-0.030)	0.024
Attention	0.19	0.961	0.026 (0.000-0.055)	0.027
Motor	0.06	0.988	0.035 (0.000-0.056)	0.028
Hyperactivity	0.15	0.993	0.031 (0.000-0.053)	0.030
Model 3: Total manganese intake from water consumption (n=375)				
Memory	0.49	1.000	0.000 (0.000-0.032)	0.027
Attention	0.11	0.950	0.032 (0.000-0.059)	0.030
Motor	0.27	0.996	0.002 (0.000-0.046)	0.030
Hyperactivity	0.10	0.991	0.030 (0.000-0.052)	0.029

CFI: comparative fit index; RMSEA: root mean square error of approximation; SRMR: standardized root mean square residual

[a]In Structural Equation Modeling (SEM), it is important to evaluate the model using several indicators because of the sensitivity of some tests to the sample size or the number of estimated parameters. We report four model fit indices that are the most insensitive to sample size, model misspecification and parameter estimates (Kline 2011), namely, the chi-square test, the RMSEA, the CFI, and the SRMR. A good model fit would provide a p-value≥0.05 for chi-square test, an RMSEA<0.06, an SRMR≤0.05, and a CFI≥0.95. All of them indicate a good to excellent model fit for all the domains except the chi-squared test for the model 1 with motor function that indicated a bad fit ($p<0.05$). The chi-squared statistic may not discriminate between good fitting models and poor fitting models because it lacks power, especially in the case of small samples such as in the present study. A detailed description of these indices and guidelines to assess model fit are provided in Hooper et al. (2008).

Table S2. Adjusted associations between manganese exposure indicators and neurobehavioral test scores, (Quebec (Canada), 2007-2009, children 6 of 13 years of age).

Neurobehavioral test scores[a]	Hair Mn β (95% CI)	Water Mn β (95% CI)	Manganese intake from water ingestion β (95% CI)
**Memory**			
CVLT, List A total trials 1-5 free recall	-3.9 (-5.6, -2.1)**	-1.0 (-1.8, -0.2)**	-0.4 (-0.9, 0.2)
CVLT, List A, trial 1 free recall	-0.8 (-1.2, -0.4)**	-0.1 (-0.3, 0.1)	-0.1 (-0.2, 0.1)
CVLT, List A, trial 5 free recall	-1.0 (-1.4, -0.6)**	-0.3 (-0.5, -0.1)**	-0.1 (-0.3, 0.0)*
CVLT, Short delay free recall	-1.4 (-1.9, -0.8)**	-0.4 (-0.7, -0.2)**	-0.2 (-0.4, -0.0)**
CVLT, Long delay free recall	49.5 (-87.6, 186.7)	-24.4 (-79.5, 30.8)	-4.8 (-45.0, 35.4)
Digit Span forward	-0.2 (-0.5, 0.2)	0.0 (-0.2, 0.2)	0.0 (-0.1, 0.1)
Digit Span backward	-0.2 (-0.4, 0.1)	-0.1 (-0.2, 0.0)*	0.0 (-0.1, 0.1)
**Attention**			
CPT-II, Omissions	-0.7 (-4.7, 3.4)	-0.1 (-1.8, 1.7)	-0.8 (-2.1, 0.4)
CPT-II, HitRT	-6.1 (-8.8, -3.4)**	-0.8 (-2.0, 0.4)	-0.2 (-1.0, 0.7)
CPT-II, Beta	-2.9 (-5.1, -0.7)**	0.1 (-0.8, 1.0)	0.1 (-0.6, 0.8)
**Motor**			
Fingertapping, dominant hand	0.3 (-2.0, 2.7)	1.2 (-0.5, 4.4)	0.0 (-1.7, 1.8)
Fingertapping, non-dominant hand	0.0 (-1.9, 1.9)	0.9 (-1.2, 3.1)	-0.1 (-1.6, 1.5)
Santa Ana, dominant hand	-0.9 (-2.3, 0.5)	-0.8 (-1.4, -0.2)**	-0.7 (-1.1, -0.2)**
Santa Ana, non-dominant hand	-0.8 (-2.0, 0.4)	-0.6 (-1.1, 0.0)*	-0.4 (-0.8, -0.0)**
**Hyperactivity**			
CRS-Parental, Hyperactivity	-2.1 (-4.4, 0.2)*	-0.3 (-1.4, 0.8)	0.2 (-0.6, 1.0)
CRS-Parental, DSM-IV: hyperactivity-impulsivity	-1.7 (-4.1, 0.3)*	-0.2 (-1.2, 0.8)	0.1 (-0.6, 0.8)
CRS-Teacher, Hyperactivity	-1.8 (-4.1, 0.5)	0.0 (-1.0, 1.1)	0.0 (-0.7, 0.7)
CRS-Teacher, DSM-IV: hyperactivity-impulsivity	-1.9 (-4.3, 0.5)	0.1 (-0.9, 1.2)	0.0 (-0.8, 0.8)

*p<0.1; **p<0.05

[a]Attention test scores were reversed; thus, higher scores for memory, attention, and motor test scores indicate better performance, but higher scores for hyperactivity suggest more problems related to hyperactivity.

All models were adjusted for child's sex, age, maternal education, non-verbal maternal intelligence, family income, maternal depressive symptoms, and lead levels in drinking water.

References

Bouchard MF, Sauve S, Barbeau B, Legrand M, Brodeur ME, Bouffard T, et al. 2011. Intellectual impairment in school-age children exposed to manganese from drinking water. Environ Health Perspect 119:138-143.

Hooper D, Coughlan J, Mullen M. 2008. Structural equation modelling: guidelines for determining model fit. EJBRM 6:53-60.

Kline R. 2011. Principles and practice of structural equation modeling. Third edition. New York: Guilford Press.
